# Supplementary material for: The burden and care cascade in young and middle-aged patients with diabetes hypertension comorbidity with abdominal obesity in India: A nationally representative cross-sectional survey
Source: PLOS Glob Public Health. 2024 Jul 17;4(7):e0003413. doi: 10.1371/journal.pgph.0003413 (PMC11253957; doi:10.1371/journal.pgph.0003413)
Supplement: S1 Table — (DOCX) [file pgph.0003413.s001.docx]

**S1 Table: Diagnostic Criteria of Metabolic Syndrome as per NCEP ATP-III**

| **Component** | **Defining level** |
| --- | --- |
| **Waist circumference** | Males ≥40 inches  Females ≥35 inches |
| **Triglycerides*** | ≥150 mg/dl |
| **HDL cholesterol*** | Males <40 mg/dl  Females <50 mg/dl |
| **Hypertension** | Systolic ≥130 mmHg or Diastolic ≥85mmHg or current use of anti-hypertensive drugs |
| **Hyperglycemia** | Fasting blood sugar ≥100 mg/dl or current use of anti-diabetes drugs |

*Data not available for present study
