# Supplementary material for: The burden and care cascade in young and middle-aged patients with diabetes hypertension comorbidity with abdominal obesity in India: A nationally representative cross-sectional survey
Source: PLOS Glob Public Health. 2024 Jul 17;4(7):e0003413. doi: 10.1371/journal.pgph.0003413 (PMC11253957; doi:10.1371/journal.pgph.0003413)
Supplement: S2 Table — (DOCX) [file pgph.0003413.s002.docx]

**S2 Table: Descriptive Statistics for Continuous Variables**

| **Variables** | **Mean (Standard deviation)** |
| --- | --- |
| **Age (years)** | 30.64 (9.99) |
| **BMI (kg/m^2^)** | 22.45 (4.62) |
| **Systolic blood pressure (mmHg)** | 116.15 (14.12) |
| **Diastolic blood pressure (mmHg)** | 77.59 (9.71) |
| **Random blood sugar (mg/dL)** | 112.99 (30.42) |
| **Waist circumference (cms)** | 78.06 (12.67) |
