# Supplementary material for: The burden and care cascade in young and middle-aged patients with diabetes hypertension comorbidity with abdominal obesity in India: A nationally representative cross-sectional survey
Source: PLOS Glob Public Health. 2024 Jul 17;4(7):e0003413. doi: 10.1371/journal.pgph.0003413 (PMC11253957; doi:10.1371/journal.pgph.0003413)
Supplement: Attachment — Submitted filename: PGPH-D-23-01679.pdf [file pgph.0003413.s003.pdf]

PLOS Global Public Health

The burden and care cascade in young and middle-aged patients with Diabetes hypertension comorbidity with abdominal obesity in India: a nationally representative cross-sectional survey

--Manuscript Draft--

Manuscript Number:	PGPH-D-23-01679
Article Type:	Research Article
Full Title:	The burden and care cascade in young and middle-aged patients with Diabetes hypertension comorbidity with abdominal obesity in India: a nationally representative cross-sectional survey
Short Title:	Burden and care in patients with Diabetes hypertension and obesity in India
Corresponding Author:	Kara Barzangi<br>University of Cambridge School of Clinical Medicine<br>Cambridge, UNITED KINGDOM
Order of Authors:	Saurav Basu
	Vansh Maheshwari
	Mansi Malik
	Kara Barzangi
	Refaat Hassan
Keywords:	Metabolic Syndrome; adults; diabetes; hypertension; abdominal obesity; India; NFHS; NCEP
Abstract:	Background<br><br>We ascertained the burden, determinants, and care cascade in the young and middle-aged patients having co-existing hypertension (HTN), Diabetes Mellitus (DM), and abdominal obesity in India from a secondary data analysis of nationally representative data.<br><br>Methods<br><br>The study examined information from the National Family Health Survey (NFHS-5) conducted in India from 2019 to 2021 in 788974 individuals aged 15-49 years including 695707 women and 93267 men.<br><br>Results<br><br>The weighted prevalence of DM-HTN comorbidity with high waist circumference in the sample was 0.75% (95% CI: 0.71 to 0.79) including 46.33% (95% CI: 44.06 to 48.62) newly diagnosed cases detected for HTN and high blood sugars. The weighted prevalence of Metabolic syndrome as per NCEP ATPIII criteria was found to be 1.13% (95% CI: 1.08 to 1.17). Only 46.16% existing cases were treated with both anti-diabetes and antihypertensive medication (full treatment), while 34.71% cases were untreated. On adjusted analysis, increasing age, females, higher wealth index, high fat diet, obesity and comorbidities were significantly associated with higher odds of having DM-HTN comorbidity with high-waist circumference.<br><br>Conclusions<br><br>At least 6 in 10 young and middle aged-population in India with DM-HTN-abdominal obesity triad are not initiated on treatment for DM and HTN comorbidities, while a majority of the previously diagnosed cases have uncontrolled blood pressure and poor glycemic control. The poor cascade of care for DM and HTN in these high-risk group of patients may substantially increase their risk for early progression and severity of microvascular and macrovascular complications especially cardiovascular disease.

Powered by Editorial Manager® and ProduXion Manager® from Aries Systems Corporation

Additional Information:	
**Question**	**Response**
**Financial Disclosure**<br><br>Enter a financial disclosure statement that describes the sources of funding for the work included in this submission and the role the funder(s) played. This includes grants and any commercial funding of the work or authors.<br><br>This statement will be typeset if the manuscript is accepted for publication.<br><br>*Please review the submission guidelines and the instructions link below for detailed requirements and guidance.*	This research received no specific grant from any funding agency in the public, commercial, or not-for-profit sectors.
**Competing Interests**<br><br>On behalf of all authors, disclose any competing interests that could be perceived to bias this work.<br><br>This statement will be typeset if the manuscript is accepted for publication.<br><br>*Please review the instructions link below and PLOS Global Public Health's competing interests policy to determine what information must be disclosed at submission.*	The authors declare that they have no known competing financial interests or personal relationships that could have appeared to influence the work reported in this paper.
**Data Availability**<br><br>Before publication, Authors are required to make fully available and without restriction all data underlying their findings. Please see our PLOS Data Policy page for detailed information on this policy.<br><br>A **Data Availability Statement**, detailing	The data is available on request from the DHS program.

where the data can be accessed, is required at first submission. Insert your Data Availability Statement in the box below.<br><br>Please see the data reporting section of our submission guidelines for instructions on what you need to include in your Data Availability Statement.<br><br>This statement will be typeset if the manuscript is accepted for publication.<br><br>*PLOS allows rare exemptions to address legal and ethical concerns. If you have legal or ethical restrictions, please use the box below to detail these in full sentences for the Journal team to consider.*	

Powered by Editorial Manager® and ProduXion Manager® from Aries Systems Corporation

Manuscript Click here to access/download;Manuscript;Manuscript - 18 August 2023 Final (1).docx

TITLE

The burden and care cascade in young and middle-aged patients with Diabetes hypertension comorbidity with abdominal obesity in India: a nationally representative cross-sectional survey

AUTHORS

Saurav Basu1 (saurav.basu@iiphd.org), Vansh Maheshwari1 (vanshmaheshwari00@outlook.com), Mansi Malik1 (mansidr676@gmail.com), Kara Barzangi2*, Refaat Hassan2 (rh684@cantab.ac.uk)

1 = Indian Institute of Public Health - Delhi, Public Health Foundation of India

2 = University of Cambridge, School of Clinical Medicine

* Corresponding Author

ABSTRACT

Background:

We ascertained the burden, determinants, and care cascade in the young and middle-aged patients having co-existing hypertension (HTN), Diabetes Mellitus (DM), and abdominal obesity in India from a secondary data analysis of nationally representative data.

Methods:

The study examined information from the National Family Health Survey (NFHS-5) conducted in India from 2019 to 2021 in 788974 individuals aged 15-49 years including 695707 women and 93267 men.

Results:

The weighted prevalence of DM-HTN comorbidity with high waist circumference in the sample was 0.75% (95% CI: 0.71 to 0.79) including 46.33% (95% CI: 44.06 to 48.62) newly diagnosed cases detected for HTN and high blood sugars. The weighted prevalence of Metabolic syndrome as per NCEP ATPIII criteria was found to be 1.13% (95% CI: 1.08 to 1.17). Only 46.16% existing cases were treated with both anti-diabetes and antihypertensive medication (full treatment), while 34.71% cases were untreated. On adjusted analysis, increasing age, females, higher wealth index, high fat diet, obesity and comorbidities were significantly associated with higher odds of having DM-HTN comorbidity with high-waist circumference.

Conclusions:

At least 6 in 10 young and middle aged-population in India with DM-HTN-abdominal obesity triad are not initiated on treatment for DM and HTN comorbidities, while a majority of the previously diagnosed cases have uncontrolled blood pressure and poor glycemic control. The poor cascade of care for DM and HTN in these high-risk group of patients may substantially increase their risk for early progression and severity of microvascular and macrovascular complications especially cardiovascular disease.

Keywords:

Metabolic Syndrome, adults, diabetes, hypertension, abdominal obesity, India, NFHS, NCEP

1. INTRODUCTION

The Global Action Plan for the Prevention and Control of Noncommunicable Diseases (NCDs), aims to halt by 2025 the increasing burden of diabetes mellitus (DM) and reduce the relative prevalence of hypertension (HTN) by 25% [1]. NCDs account for 71% of the total global mortality and 15 million premature deaths before the age of 70 years, with the WHO setting the goal of reducing premature mortality from NCDs by one-third by 2030 [1,2].

The number of deaths from NCDs in India in 2017 was estimated to be around 4.7 million, accounting for 49% of all-cause mortality. The leading causes of these deaths were cardiovascular diseases (23%), chronic respiratory diseases (9%), cancer (6%) and diabetes (2.4%) with 65% of these deaths being premature and preventable [3]. The estimated percentage of disability-adjusted life years (DALYs) in India attributable to NCDs has increased from 30% of all DALYs in 1990 to 58% in 2019 [4].

Leading risk factors driving the burden of cardiovascular diseases, the single biggest cause of mortality worldwide include diabetes mellitus (DM), hypertension (HTN), and obesity [5]. As of 2021, 74 million individuals live in India with DM and this burden expected to reach 125 million by 2045 [6]. Additionally, Hypertension, a major modifiable risk factor for cardiovascular disease (CVD), has an estimated prevalence in 24% of men and 21% of women as per a nationally representative cross-sectional survey in the 15-49 aged population of India [7,8].

Abdominal obesity is a condition of excessive fat deposition around the abdominal region signifying insulin resistance and ectopic fat accumulation with elevated adipokine levels that substantially increases the risk of multiple adverse health conditions including T2DM, hypertension, CVD, nephrotic syndrome, and even cancer [9].

The presence of Metabolic syndrome as per the WHO requires the presence of any one of DM, impaired fasting glucose or insulin resistance, impaired glucose tolerance and at least two of the following conditions: hypertension, central obesity, dyslipidaemia and high creatinine-albumin ratio. Consequently, the co-existence of the above three conditions (diabetes, hypertension and abdominal obesity) in an individual fulfils the diagnostic criteria for Metabolic Syndrome [10].

A recently published systematic review estimated 30% prevalence of Metabolic syndrome among the adult population in India with six times higher odds of the condition in obese individuals [11,12]. Regardless of prior histories of cardiovascular events, people with Metabolic syndrome have an average four-fold increased risk of getting a stroke or myocardial infarction and a two-fold increased risk of dying from a comparable event [13].

Addressing the challenge of Metabolic syndrome in India is essential towards reducing the risk of NCD related complications with high morbidity, mortality, and associated socioeconomic health costs. Lifestyle modifications through healthy diet and exercise can reverse prediabetes, early-stage hypertension, and abdominal obesity thereby reducing the risk of its complications [14,15]. Furthermore, in existing patients with DM and/or HTN with abdominal obesity, adherence to medications apart from healthy lifestyle and self-care is crucial for maintaining controlled blood glucose and reduced blood pressure levels that reduce the risk of complications [16,17]. However, evidence from national level surveys from India suggest that most patients with DM and/or HTN fail to achieve optimal blood pressure and glycemic control that is associated with reduced quality of life and poor health outcomes [18,19]. Patients with multimorbidity conditions as in Metabolic syndrome are subjected to complex pharmacological regimes that is associated with reduced medication adherence due to polypharmacy and regimen complexity and potential drug interactions and associated side effects [20,21].

Care cascade for a chronic disease indicates the sequential steps a person would take from the stages of screening, diagnosis, treatment initiation, and achievement of target health outcomes. Analysis of these cascade can help to generate quantitative evidence on the gaps in the existing framework for delivery of care and the areas of concern where quality of care warrant improvement [22]. However, till date, evidence from nationally representative survey data has not been utilized to assess the quality and effectiveness of the care cascade especially in young and middle-aged patients with Metabolic syndrome in India.

We therefore conducted this study with the objective of ascertaining the burden, determinants, and care cascade in the young and middle-aged patients having co-existing HTN, DM, and abdominal obesity in India.

2. METHODS

2.1 Data Source:

The study examined information from the National Family Health Survey (NFHS-5) conducted in India from 2019 to 2021. A two-stage stratified sample is utilized in the NFHS-5. The probability proportional to size (PPS) sampling method was used to select the primary sample units (PSUs), which were villages in rural areas and Census Enumeration Blocks (CEBs) in urban areas. This study analysed data from men and women aged 18-49 years who consented to blood pressure and random blood sugar measurements [8].

2.2 Sample size:

The study's sample size consisted of 788974 individuals aged 15-49 years (695707 women and 93267 men).

2.3 Outcome variables:

(1) Diabetes-hypertension comorbidity along with high waist circumference was the primary outcome variable of this study. Cases of DM and HTN included both previously diagnosed cases and newly diagnosed cases.

(2) High-risk categories of DM-HTN-High waist circumference were considered as those individuals who had suboptimal glycemic (random blood sugar measurement ≥180 mg/dl) and suboptimal blood pressure control (≥140/90 mmHg) as per the JNC-7 criteria [23].

2.4 Independent variables:

Covariates included age, sex, employment status, educational attainment, smoking status, level of physical activity, and marital status (individual-level variables) and wealth status, media access, place of residence, and region (household level variables).

(1) Body mass index (BMI) was defined as: Underweight (<18.5 kg/m2), Normal (18.5–24.9 kg/m2), Overweight (25.0–29.9 kg/m2) and Obese (≥30.0 kg/m2) [24].

(2) Physical Activity Levels: Information on physical activity was not directly available in the NFHS-5 dataset. Hence, occupation was considered as a surrogate to assess the physical activity levels. Any respondent whose work responsibilities involved physical activities were regarded as 'involved in an occupation with high physical activity'; otherwise, they were considered to 'involve less physical activity' i.e., Not working, clerical and sales jobs = involve less physical activity and other job types = involve high physical activity [25].

(3) Diet: was categorized into a high and low-fat diet based on previous literature [26,27]. The high-fat diet consisted of individuals who said 'Yes' to either "Daily eggs", "Daily fish", "Daily chicken or meat" or "Daily fried foods" consumption. Whereas, the remaining participants were put in the Low-fat diet category.

(4) Additional Comorbidities: This was treated as a dichotomous variable where respondents were assigned to the 'No comorbidity' group if they responded 'no' to all the following questions:

do you currently have any heart disease?; do you currently have chronic respiratory diseases including asthma?; do you currently have a goitre or any other thyroid disorder?; do you currently have chronic kidney disease? If the participant responded 'yes' to any one or more of the questions above, then they were grouped in the "one or more comorbidity" group.

(5) Media Exposure:

If the respondent said 'no' to the question: "Owns a mobile telephone" and said "not at all" to the question: "frequency of watching television/listening to the radio/ reading the newspaper" then the participant was considered to have "no media exposure". If the respondent said 'yes' to any of the questions mentioned above, then the participant was considered to "have media exposure".

2.5 Operational Definitions:

(1) Previously diagnosed patients with DM: Those who said yes to "told they had high glucose levels on two or more occasions by a doctor or other health professional", or taking medications to lower blood glucose levels were considered as previously diagnosed.

(2) Newly diagnosed patients with DM: Individuals who were not fasting and had Random Blood Sugar (RBS) levels ≥200 mg/dl or ≥126 mg/dl if fasting for ≥8 hours during the survey, and responded ‘no’ to the following two questions:

1. Told they had high glucose on two or more occasions by the doctor.

2. Currently taking prescribed medicine to lower glucose levels.

(3) Previously diagnosed patients with Hypertension: Those who said yes to “told they had high bp on two or more occasions by a doctor or other health professional”, or taking medications to lower blood pressure were considered as previously diagnosed.

(4) Newly diagnosed patients with Hypertension: Individuals who had an average of the last two blood pressure readings (BP) ≥140/90 mmHg on screening during the survey and responded ‘no’ to the following two questions:

1. Told they had high BP on two or more occasions by the doctor.

2. Currently taking prescribed medicine to lower BP.

(5) Abdominal Obesity: Present if waist circumference ≥35 inches for women and ≥40 inches for men.

(6) On Diabetes Treatment: Individuals who responded ‘yes’ to the following question: “Currently taking a prescribed medicine to lower blood glucose”, were considered to be on treatment for hypertension.

(7) On Hypertension Treatment: Individuals who responded ‘yes’ to the following question: “Currently taking a prescribed medicine to lower blood pressure”, were considered to be on treatment for diabetes.

(8) Full treatment was considered as the previously diagnosed cases of DM and HTN initiated on both anti-diabetes and antihypertensive treatment

(9) Partial treatment was considered as the previously diagnosed cases of DM and HTN initiated on either anti-diabetes or antihypertensive treatment but not both.

(10) Metabolic syndrome was defined as per National Cholesterol Education Program (NCEP) Adult Treatment Panel III (ATP III) [28] as the presence of three or more of the following five criteria: waist circumference over 40 inches (men), or 35 inches (women), blood pressure over 130/85 mmHg, fasting triglyceride levels (TG) over 150 mg/dl, fasting High-density lipoprotein (HDL) cholesterol level <40 mg/dl (men), or 50 mg/dl (women), and fasting blood sugar over 100 mg/dl. Since, data on TG and HDL was not available in the NFHS, we defined metabolic syndrome as the presence of the remaining 3 conditions.

2.6 Methodology:

For blood glucose testing, the Accu-Chek Performa glucometer with glucose test strips was used to collect finger-stick blood samples. A previously undiagnosed individual was considered to have diabetes if the RBS ≥200mg/dl.

Blood pressure was measured for all women and men aged 15 and above using an Omron Blood Pressure Monitor to determine the prevalence of hypertension. Blood pressure measurements for each respondent were taken three times with an interval of five minutes between readings [8]. The average of the second and third measurements was taken and participants with SBP of ≥140 mmHg and/or a DBP of ≥90 mmHg were regarded as hypertensive, while those already on antihypertensive medicines to regulate their BP were also considered hypertensive [23].

The waist circumference (WC) is a validated measurement of abdominal obesity [29]. The waist circumference measurement for assessment of abdominal obesity were done by using Gulick tapes [8].

2.7 Statistical Analysis:

Categorical variables were reported as frequencies and proportions. Continuous variables were reported as means and standard deviations (SD). Bivariate analysis was performed and the chi-square test was used to identify the relationship between dependent and predictor variables. Variables

that were statistically significant in the bivariate analysis were put in the adjusted multivariable logistic regression model. The model assumptions regarding linearity in the logit function, multicollinearity, and the presence of outliers were assessed.

Furthermore, to evaluate the treatment seeking behaviour of previously diagnosed DM-HTN comorbid patients having high waist circumference, the treatment behaviour was categorized into three groups: No treatment for both DM and HTN, partial treatment (i.e., treatment for either DM or HTN), and full treatment (for both DM and HTN). We applied multinomial regression model to estimate the regression coefficients for all three treatment behaviours. Variables found to be significantly associated in the unadjusted model were included in the adjusted multinomial model. We utilized the modified Poisson regression approach [30] for assessing the determinants of previously diagnosed, incident and prevalent DM-HTN comorbid cases with high-waist circumference. The model was fit using a Generalized Linear Model (GLM) with the Poisson family and a robust error variance. Model fit was checked by Akaike Information Criterion (AIC) and the Bayesian Information Criterion (BIC). A variable was considered to be statistically significant if the P-value was less than 0.05. The "svy" suffix was applied to perform weighted analysis to regulate the clustering effect. Data analysis was conducted using Stata version 15.1 (StataCorp, College Station, TX, USA).

2.8 Ethics approval:

The ethical approval for NFHS 5 survey was obtained from ethics review board of the International Institute of Population Sciences, Mumbai, India. Written and informed consent was obtained from each respondent before participating in the survey, additionally, written informed consent was obtained from the parent/guardian of each respondent under 18 years of age. No separate ethical approval was required for this secondary data analysis, since the NFHS-5 dataset is an anonymous publicly available dataset with no identifiable information about the study participants. The data were accessed on the 15th of February 2023 for research purposes, and authors never had access to information that could identify individual participants during or after data collection.

3. RESULTS

We included 788974 individuals aged 15-49 years (695707 women and 93267 men) in our analysis. The weighted mean (SD) age of the participants was 30.64 (9.99) years. The weighted prevalence of DM-HTN comorbidity with high waist circumference was 0.75% (n = 4644, 95% CI: 0.71 to 0.79) in the sample including 46.33% (n = 2070, 95% CI: 44.06 to 48.62) newly diagnosed cases detected on screening for HTN and high blood sugars. Among the participants (N = 274256) at high risk of metabolic syndrome (either having DM/HTN/high waist/BMI≥25), the weighted prevalence of DM-HTN-high waist circumference was 2.00% (n = 4644, 95% CI: 1.91 to 2.09) including 0.93% (n = 2070, 95% CI: 0.87 to 0.99) newly diagnosed cases. The weighted prevalence of Metabolic syndrome as per NCEP ATPIII criteria in the participants was 1.13% (n = 6951, 95% CI: 1.08 to 1.17).

Table 1 reports the socio-demographic and lifestyle characteristics among the participants having DM-HTN comorbidity with high waist circumference. Among the previously diagnosed cases, a majority of them were middle-aged (41-49 years) (59.12%) and females (96.18%).

Among the previously diagnosed cases of DM-HTN comorbidity with high waist circumference, 46.16% (95% CI: 43.02 to 49.32) cases were treated with both anti-diabetes and antihypertensive medication (full treatment), 19.14% (95% CI: 17.12 to 21.33) cases were on either anti-diabetes or antihypertensive medication but not both (partial treatment), while 34.71% (95% CI: 31.70 to 37.84) cases were on neither treatment (untreated). Table 2 reports the treatment seeking behaviour of previously diagnosed DM-HTN comorbid patients having high waist circumference. Upon unadjusted multinomial regression analysis, males and increasing age were significantly associated with the odds of availing partial or full treatment for DM-HTN comorbidity. Similarly, higher wealth index, urban residency, increasing BMI, exposure to media and presence of additional comorbidities were positively associated with availing full treatment as compared to availing no treatment. Adjusted analysis showed that individuals belonging to age group 41-49 years (aRRR = 3.19, 95% CI: 1.96 to 5.20) and richest wealth index (aRRR = 2.81, 95% CI: 1.36 to 5.81) were more likely to be on partial treatment rather than not taking any treatment. Similarly, individuals having age 41-49 years (aRRR = 18.03, 95% CI: 9.87 to 32.94), richest wealth index (aRRR = 2.42, 95% CI: 1.14 to 5.11), urban residency (aRRR = 1.62, 95% CI: 1.15 to 2.27),

obesity (aRRR = 1.56, 95% CI: 1.02 to 2.37), media exposure (aRRR = 1.52, 95% CI: 1.03 to 2.55) and additional comorbidities (aRRR = 1.60, 95% CI: 1.16 to 2.22) were more likely to be on full treatment as compared to not being on either treatment. Further, females (aRRR = 0.42, 95% CI: 0.20 to 0.87) were less likely to be on full treatment than no treatment as compared to males.

Table 3 reports the association of the socio-demographic and lifestyle characteristics among both the cases of DM-HTN comorbidity with high-waist circumference stratified by their time of diagnosis (previous, new, total). On adjusted analysis, increasing age, female sex, higher wealth index, increasing BMI and exposure to media had significantly higher odds of having a previous diagnosis of DM-HTN comorbidity with high-waist circumference. Similarly, increasing age, lower education levels, urban residence, higher wealth index and high BMI were the predictors associated with newly diagnosed cases of DM-HTN comorbidity with high-waist circumference. The risk factors that were significantly associated with occurrence of DM-HTN-comorbidity with high waist circumference in the total cases (both previously diagnosed and new cases) were the middle age group (41-49 years) compared to younger aged cases (aRR = 6.12, 95% CI: 5.24 to 7.15), females compared to male cases (aRR = 1.62, 95% CI: 1.25 to 2.10), higher compared to no education (aRR = 0.84, 95% CI: 0.71 to 0.99), cases belonging to richest wealth index compared to lower wealth quintiles (aRR = 1.91, 95% CI: 1.52 to 2.40), obesity (aRR = 4.68, 95% CI: 4.06 to 5.40), high fat diet compared to low fat diet (aRR = 1.18, 95% CI: 1.06 to 1.30) and presence of additional comorbidities compared to no other self-reported comorbidities (aRR = 1.47, 95% CI: 1.32 to 1.63).

Furthermore, less than half (46.83%) of the cases with DM-HTN comorbidity with high waist circumference had both controlled BP (<140/90) and blood glucose levels (RBS<180). However, nearly 18% of the cases had both suboptimal blood pressure and glycemic control (Figure 1). Regional variation was observed in the prevalence of DM-HTN comorbidity with high waist circumference across Indian states and union territories with Chandigarh having the highest prevalence (2.53%), followed by Tamil Nadu (2.31%), while the lowest was observed in Meghalaya (0.12%) (Figure 2).

4. DISCUSSION

The present study observed the prevalence of DM-HTN comorbidity with high waist circumference equivalent to metabolic syndrome (Metabolic syndrome) was lower (0.75%) compared to regional estimates of metabolic syndrome [31,32] although it is an underestimation as the case definitions in this study did not account for elevated triglyceride or HDL-C levels which were unavailable, and also the lack of older adults and elderly in the sample.

The overall prevalence of DM-HTN comorbidity with high waist circumference was significantly higher in females, a finding consistent with the findings of a meta-analysis of studies from India. [11]. The presence of Metabolic syndrome in this study was also associated with increasing age and higher BMI, findings that are consistent with the global evidence [33–35]. Furthermore, in this study, people living in urban areas were 1.2 times more likely to have Metabolic syndrome as compared to rural residents suggestive of the linkage of adverse social determinants such as sedentary lifestyle, dietary changes and also stress in urban areas [33].

The present study findings indicate that higher education levels decrease the risk of having undiagnosed metabolic syndrome in accordance with a study conducted in China [36], suggestive of the impact of adverse social determinants of health contributed to delayed screening and diagnosis of DM and HTN in individuals with lower educational status compared with their counterparts. Less than half of the cases in our sample (46.8%) having DM-HTN comorbidity with high waist circumference and previously initiated on treatment had poorly controlled blood pressure and blood glucose levels that may reflect either poor medical adherence, or poor response to hypertension treatment [37] and insulin resistance [38]. Furthermore, patients with Metabolic syndrome satisfying criteria of having high waist circumference, elevated blood pressure and triglyceride levels require early and effective treatment to prevent the onset and progression of cardiovascular complication [39]. However, in this study, nearly one in two cases with Metabolic syndrome were undiagnosed that were detected on survey-based screening of blood pressure and blood glucose levels signifying an iceberg phenomenon of disease wherein only a fraction of the total cases is clinically diagnosed and initiated on effective treatment. Consequently, public health strategies to control the pandemic of Metabolic syndrome driven cardiovascular disease burden in the developing world including India requires scaling up of screening individuals above age 30 especially those with any high-risk factor for Metabolic syndrome for HTN, DM, abdominal obesity, and elevated blood triglyceride levels. Furthermore, initiation of effective treatment for cases

diagnosis with HTN, DM, or Metabolic syndrome is of paramount significance. In this context, the revamped national program for non-communicable diseases (NP-NCD 2023-2030) in India has set an ambitious 75:25 initiative for placing 75 million patients with HTN or DM on standard by 2025 [40].

The study has certain strengths including its analysis of a large sample size and nationally representative dataset with adequate geographical diversity that supports generalizability of the findings. However, there are also some major study limitations. First, the cross-sectional design of the study does not permit assessment of temporal association and precludes causal assessment. Second, information for two important components of metabolic syndrome, serum cholesterol and triglyceride levels were not available in the NFHS datasets that is likely to have contributed to significant underestimation of the burden of the problem. Third, there may be some recall bias, although blood pressure and random blood sugar measurements were conducted as part of the study. However, the lack of fasting blood glucose and glycated haemoglobin measurements precluded the validated estimation of glycemic control in the patients. Lastly, no direct information on physical activity was available in the datasets so a surrogate was obtained from the individual's occupation which may not accurately reflect the physical activity status of the participants.

In conclusion, nearly 6 in 10 young and middle aged-population in India with DM-HTN-abdominal obesity triad are not initiated on treatment for DM and HTN comorbidities especially those from socioeconomically disadvantaged groups, while a majority of the previously diagnosed cases have uncontrolled blood pressure and poor glycemic control. The poor cascade of care for DM and HTN in these high-risk group of patients may substantially increase their risk for early progression and severity of microvascular and macrovascular complications especially cardiovascular disease. Early diagnosis of cases of metabolic syndrome including DM and HTN, with early initiation of effective medication and treatment, regular monitoring and targeted reduction of blood pressure, blood glucose, and body weight through primary health system strengthening and community engagement remain the cornerstone of reducing complications from these debilitating health conditions.

5. DECLARATIONS

Conflicts of interest = None

Funding = This research received no specific grant from any funding agency in the public, commercial, or not-for-profit sectors

Data availability = The data is available on request from the DHS program

Acknowledgements = The authors thank DHS for providing the NFHS-5 datasets.

AUTHOR CONTRIBUTIONS

Conceptualization: SB

Methodology: All authors

Formal Analysis: VM, MM

Visualization: SB, VM, RH

Writing (Review and Editing): All authors

Writing (Original Draft): SB, VM, KB

REFERENCES

1. Global action plan for the prevention and control of NCDs 2013–2020 [Internet]. [cited 2023 May 10]. Available from: https://www.who.int/publications-detail-redirect/9789241506236

2. United Nations. World Population Prospects 2022: Summary of Results [Internet]. United Nations; 2022 [cited 2023 May 10]. (Statistical Papers - United Nations (Ser. A), Population and Vital Statistics Report). Available from: https://www.un-ilibrary.org/content/books/9789210014380

3. Menon GR, Singh L, Sharma P, Yadav P, Sharma S, Kalaskar S, et al. National Burden Estimates of healthy life lost in India, 2017: an analysis using direct mortality data and indirect disability data. Lancet Glob Health. 2019 Dec;7(12):e1675–84.

4. GBD Compare [Internet]. Institute for Health Metrics and Evaluation. [cited 2023 May 10]. Available from: http://vizhub.healthdata.org/gbd-compare

5. The India State-Level Disease Burden Initiative - Public Health Foundation of India [Internet]. 2018 [cited 2023 May 10]. Available from: https://phfi.org/the-work/research/the-india-state-level-disease-burden-initiative/

6. Home, Resources, diabetes L with, Acknowledgement, FAQs, Contact, et al. IDF Diabetes Atlas | Tenth Edition [Internet]. [cited 2023 May 10]. Available from: https://diabetesatlas.org/

7. Roth GA, Mensah GA, Johnson CO, Addolorato G, Ammirati E, Baddour LM, et al. Global Burden of Cardiovascular Diseases and Risk Factors, 1990–2019. J Am Coll Cardiol. 2020 Dec 22;76(25):2982–3021.

8. National Family Health Survey (NFHS-5) INDIA Report | International Institute for Population Sciences (IIPS) [Internet]. [cited 2023 May 10]. Available from: https://iipsindia.ac.in/content/national-family-health-survey-nfhs-5-india-report

9. Dhawan D, Sharma S. Abdominal Obesity, Adipokines and Non-communicable Diseases. J Steroid Biochem Mol Biol. 2020 Oct;203:105737.

10. Alberti KG, Zimmet PZ. Definition, diagnosis and classification of diabetes mellitus and its complications. Part 1: diagnosis and classification of diabetes mellitus provisional report of a WHO consultation. Diabet Med. 1998 Jul;15(7):539–53.

11. Krishnamoorthy Y, Rajaa S, Murali S, Rehman T, Sahoo J, Kar SS. Prevalence of metabolic syndrome among adult population in India: A systematic review and meta-analysis. PLOS ONE. 2020 Oct 19;15(10):e0240971.

12. Krishnamoorthy Y, Rajaa S, Murali S, Sahoo J, Kar SS. Association between behavioural risk factors and metabolic syndrome among adult population in India: A systematic review and meta-analysis of observational studies. Nutr Metab Cardiovasc Dis. 2022 Jan;32(1):40–52.

13. Martín-Timón I, Sevillano-Collantes C, Segura-Galindo A, del Cañizo-Gómez FJ. Type 2 diabetes and cardiovascular disease: Have all risk factors the same strength? World J Diabetes. 2014 Aug 15;5(4):444–70.

14. Rhee E, Cho J, Kwon H, Park S, Jung J, Han K, et al. Association between abdominal obesity and increased risk for the development of hypertension regardless of physical activity: A nationwide population- based study. J Clin Hypertens (Greenwich). 2018 Oct 13;20(10):1417–26.

15. Galaviz KI, Weber MB, Suvada K, Gujral UP, Wei J, Merchant R, et al. Interventions for Reversing Prediabetes: A Systematic Review and Meta-Analysis. Am J Prev Med. 2022 Apr;62(4):614–25.

16. Choudhry NK, Kronish IM, Vongpatanasin W, Ferdinand KC, Pavlik VN, Egan BM, et al. Medication Adherence and Blood Pressure Control: A Scientific Statement From the American Heart Association. Hypertension. 2022 Jan;79(1):e1–14.

17. Huang J, Ding S, Xiong S, Liu Z. Medication Adherence and Associated Factors in Patients With Type 2 Diabetes: A Structural Equation Model. Front Public Health. 2021;9:730845.

18. Koya SF, Pilakkadavath Z, Chandran P, Wilson T, Kuriakose S, Akbar SK, et al. Hypertension control rate in India: systematic review and meta-analysis of population-level non-interventional studies, 2001–2022. The Lancet Regional Health - Southeast Asia. 2023 Feb;9:100113.

19. Mathur P, Leburu S, Kulothungan V. Prevalence, Awareness, Treatment and Control of Diabetes in India From the Countrywide National NCD Monitoring Survey. Front Public Health. 2022;10:748157.

20. Foley L, Larkin J, Lombard-Vance R, Murphy AW, Hynes L, Galvin E, et al. Prevalence and predictors of medication non-adherence among people living with multimorbidity: a systematic review and meta-analysis. BMJ Open. 2021 Sep 2;11(9):e044987.

21. Aggarwal P, Woolford SJ, Patel HP. Multi-Morbidity and Polypharmacy in Older People: Challenges and Opportunities for Clinical Practice. Geriatrics (Basel). 2020 Oct 28;5(4):85.

22. Subbaraman R, Nathavitharana RR, Mayer KH, Satyanarayana S, Chadha VK, Arinaminpathy N, et al. Constructing care cascades for active tuberculosis: A strategy for program monitoring and identifying gaps in quality of care. PLOS Medicine. 2019 Feb 27;16(2):e1002754.

23. National High Blood Pressure Education Program. The Seventh Report of the Joint National Committee on Prevention, Detection, Evaluation, and Treatment of High Blood Pressure [Internet]. Bethesda (MD): National Heart, Lung, and Blood Institute (US); 2004 [cited 2023 May 10]. Available from: http://www.ncbi.nlm.nih.gov/books/NBK9630/

24. Defining Adult Overweight and Obesity [Internet]. Centers for Disease Control and Prevention. 2022 [cited 2023 May 10]. Available from: https://www.cdc.gov/obesity/basics/adult-defining.html

25. Kundu S, Rahman MA, Kabir H, Al Banna MH, Hagan Jr. JE, Srem-Sai M, et al. Diabetes, Hypertension, and Comorbidity among Bangladeshi Adults: Associated Factors and Socio-Economic Inequalities. Journal of Cardiovascular Development and Disease. 2023 Jan;10(1):7.

26. Schlesinger S, Neuenschwander M, Schwedhelm C, Hoffmann G, Bechthold A, Boeing H, et al. Food Groups and Risk of Overweight, Obesity, and Weight Gain: A Systematic Review and Dose-Response Meta-Analysis of Prospective Studies. Adv Nutr. 2019 Mar 1;10(2):205–18.

27. A healthy lifestyle - WHO recommendations [Internet]. [cited 2023 May 10]. Available from: https://www.who.int/europe/news-room/fact-sheets/item/a-healthy-lifestyle---who-recommendations

28. Expert Panel on Detection, Evaluation, and Treatment of High Blood Cholesterol in Adults. Executive Summary of The Third Report of The National Cholesterol Education Program (NCEP) Expert Panel on Detection, Evaluation, And Treatment of High Blood Cholesterol In Adults (Adult Treatment Panel III). JAMA. 2001 May 16;285(19):2486–97.

29. Ross R, Neeland IJ, Yamashita S, Shai I, Seidell J, Magni P, et al. Waist circumference as a vital sign in clinical practice: a Consensus Statement from the IAS and ICCR Working Group on Visceral Obesity. Nat Rev Endocrinol. 2020 Mar;16(3):177–89.

30. Zou G. A modified poisson regression approach to prospective studies with binary data. Am J Epidemiol. 2004 Apr 1;159(7):702–6.

31. Dasgupta A, Banerjee R, Pan T, Suman S, Basu U, Paul B. Metabolic syndrome and its correlates: A cross-sectional study among adults aged 18-49 years in an Urban Area of West Bengal. Indian J Public Health. 2020;64(1):50–4.

32. Pandit VB, Jg A, P H. Metabolic Syndrome among Adult Individuals -A Preliminary Cross Sectional Study in Kurnool District. International Journal of Chemical and Lifesciences. 2013 May 1;2(5):1168–71.

33. Mehata S, Shrestha N, Mehta RK, Bista B, Pandey AR, Mishra SR. Prevalence of the Metabolic Syndrome and its determinants among Nepalese adults: Findings from a nationally representative cross-sectional study. Sci Rep. 2018 Oct 9;8(1):14995.

34. Chowdhury MZI, Anik AM, Farhana Z, Bristi PD, Abu Al Mamun BM, Uddin MJ, et al. Prevalence of metabolic syndrome in Bangladesh: a systematic review and meta-analysis of the studies. BMC Public Health. 2018 Mar 2;18:308.

35. Gu D, Reynolds K, Wu X, Chen J, Duan X, Reynolds RF, et al. Prevalence of the metabolic syndrome and overweight among adults in China. Lancet. 2005 Apr 16;365(9468):1398–405.

36. Li X, Cao C, Tang X, Yan X, Zhou H, Liu J, et al. Prevalence of Metabolic Syndrome and Its Determinants in Newly-Diagnosed Adult-Onset Diabetes in China: A Multi-Center, Cross-Sectional Survey. Front Endocrinol (Lausanne). 2019 Oct 1;10:661.

37. Zidek W, Naditch-Brûlé L, Perlini S, Farsang C, Kjeldsen SE. Blood pressure control and components of the metabolic syndrome: the GOOD survey. Cardiovasc Diabetol. 2009 Sep 15;8:51.

38. Shen H, Qi L, Tai ES, Chew SK, Tan CE, Ordovas JM. Uncoupling protein 2 promoter polymorphism -866G/A, central adiposity, and metabolic syndrome in Asians. Obesity (Silver Spring). 2006 Apr;14(4):656–61.

39. Meifang Y, Xue S, Jue H, Yina TU, Jie HE, Yiming Z, et al. [Metabolic syndrome increases Framingham risk score of patients with type 2 diabetes mellitus]. Zhejiang Da Xue Xue Bao Yi Xue Ban. 2016 May 25;45(3):268–74.

40. India: 75 million people with hypertension or diabetes on standard care by 2025 [Internet]. [cited 2023 Aug 18]. Available from: https://www.who.int/southeastasia/news/detail/18-05-2023-india-75-million-people-with-hypertension-or-diabetes-on-standard-care-by-2025

TABLES

Table 1: Socio-demographic and lifestyle characteristics of DM-HTN comorbid individuals with high waist circumference

**Characteristics**	**DM-HTN comorbid with High waist circumference**		
	**Previously diagnosed**<br>**n (weighted %)**<br>**(N = 2434)**	**Newly diagnosed**<br>**n (weighted %)**<br>**(N = 679)**	**Total**<br>**n (weighted %)**<br>**(N = 3113)**
**Age (years)**			
15-30	271 (11.13)	44 (6.48)	315 (10.12)
31-40	724 (29.75)	252 (37.11)	976 (31.35)
41-49	1439 (59.12)	383 (56.41)	1822 (58.53)
**Sex**			
Male	93 (3.82)	63 (9.28)	156 (5.01)
Female	2341 (96.18)	616 (90.72)	2957 (94.99)
**Education**			
No education	523 (21.49)	181 (26.66)	704 (22.61)
Primary	345 (14.17)	125 (18.41)	470 (15.1)
Secondary	1187 (48.77)	319 (46.98)	1506 (48.38)
Higher	379 (15.57)	54 (7.95)	433 (13.91)

**Occupation**			
With high physical activity	173 (37.28)	82 (49.4)	255 (40.48)
With low physical activity	291 (62.72)	84 (50.6)	375 (59.52)
**Place of Residence**			
Rural	1372 (56.37)	380 (55.96)	1752 (56.28)
Urban	1062 (43.63)	299 (44.04)	1361 (43.72)
**Wealth Index**			
Poorest	128 (5.26)	54 (7.95)	182 (5.85)
Poorer	267 (10.97)	106 (15.61)	373 (11.98)
Middle	460 (18.9)	158 (23.27)	618 (19.85)
Richer	692 (28.43)	177 (26.07)	869 (27.92)
Richest	887 (36.44)	184 (27.1)	1071 (34.4)
**BMI (kg/m^2)**			
Under weight	10 (0.41)	3 (0.44)	13 (0.42)
Normal	433 (17.83)	90 (13.31)	523 (16.84)
Overweight	993 (40.88)	289 (42.75)	1282 (41.29)
Obese	993 (40.88)	294 (43.49)	1287 (41.45)
**Smoking Status**			
Yes	145 (5.96)	58 (8.54)	203 (6.52)

No	2289 (94.04)	621 (91.46)	2910 (93.48)
**Alcohol consumption**			
Yes	65 (2.67)	33 (4.86)	98 (3.15)
No	2369 (97.33)	646 (95.14)	3015 (96.85)
**Media Exposure**			
Yes	2172 (89.24)	574 (84.54)	2746 (88.21)
No	262 (10.76)	105 (15.46)	367 (11.79)
**Diet**			
High fat	532 (21.86)	121 (17.82)	653 (20.98)
Low fat	1902 (78.14)	558 (82.18)	2460 (79.02)
**Additional Comorbidities**			
None	2017 (82.87)	618 (91.02)	2635 (84.65)
1 or more	417 (17.13)	61 (8.98)	478 (15.35)

Table 2: Treatment-seeking behaviour among previously diagnosed DM-HTN comorbid patients with high waist circumference

**Variables**	**Not on Treatment**<br>**n (weighted %)**<br>**(N = 1026)**	**Partial Treatment**<br>**n (weighted %)**<br>**(N = 455)**	**Full treatment**<br>**n (weighted %)**<br>**(N = 953)**	**Crude RRR[1] (95% CI)**	**Crude RRR[2] (95% CI)**	**aRRR[1] (95% CI)**	**aRRR[2] (95% CI)**
**Age (years)**							
15-30	217 (21.15)	35 (7.69)	19 (1.99)	Ref	Ref	Ref	Ref
31-40	407 (39.67)	126 (27.69)	191 (20.04)	1.92 [1.27,2.89] *	5.36 [3.25,8.83] **	1.63 [1.07,2.48] *	4.36 [2.63,7.24] **
41-49	402 (39.18)	294 (64.62)	743 (77.96)	4.53 [3.08,6.68] **	21.11 [13.00,34.26] **	3.53 [2.36,5.28] **	16.23 [9.90,26.61] **
**Sex**							
Male	18 (3.96)	44 (4.62)	93 (3.82)	Ref	Ref	-	-
Female	437 (96.04)	909 (95.38)	2341 (96.18)	0.76 [0.42,1.37]	0.64 [0.40,1.03]		
**Education**							
No education	184 (17.93)	121 (26.59)	218 (22.88)	Ref	Ref	Ref	Ref
Primary	123 (11.99)	71 (15.6)	151 (15.84)	0.88 [0.60,1.27]	1.04 [0.76, 1.41]	0.86 [0.58,1.27]	1.04 [0.74,1.45]
Secondary	507 (49.42)	206 (45.27)	474 (49.74)	0.62 [0.47,0.82] *	0.79 [0.62,1.00] *	0.61 [0.45,0.84] *	0.77 [0.58,1.01]

Higher	212 (20.66)	57 (12.53)	110 (11.54)	0.41 [0.28,0.59] **	0.44 [0.32,0.59] **	0.40 [0.26,0.61] **	0.40 [0.27,0.57] **
**Occupation**							
With higher physical activity	70 (40.46)	34 (35.42)	69 (35.38)	Ref	Ref	-	-
With lower physical activity	103 (59.54)	62 (64.58)	126 (64.62)	0.81 [0.53,1.23]	1.00 [0.60,1.66]		
**Place of Residence**							
Rural	644 (62.77)	273 (60)	455 (47.74)	Ref	Ref	Ref	Ref
Urban	382 (37.23)	182 (40)	498 (52.26)	1.12 [0.9,1.41]	1.85 [1.54,2.21] **	0.98 [0.76,1.26]	1.51 [1.22,1.88] **
**Wealth Index**							
Poorest	73 (7.12)	23 (5.05)	32 (3.36)	Ref	Ref	Ref	Ref
Poorer	149 (14.52)	43 (9.45)	75 (7.87)	0.92 [0.51,1.63]	1.15 [0.7,1.89]	0.93 [0.51,1.70]	1.00 [0.58,1.73]
Middle	211 (20.57)	82 (18.02)	167 (17.52)	1.23 [0.72,2.1]	1.81 [1.14,2.87] *	1.33 [0.76,2.34]	1.49 [0.89,2.50]
Richer	269 (26.22)	150 (32.97)	273 (28.65)	1.77 [1.06,2.95] *	2.32 [1.48,3.62] **	2.07 [1.18,3.63] *	1.80 [1.08,3.02] *
Richest	324 (31.58)	157 (34.51)	406 (42.6)	1.54 [0.93,2.55]	2.86 [1.84,4.44] **	2.00 [1.12,3.57] *	2.26 [1.34,3.81] *

**BMI (kg/m^2)**							
Under weight	6 (0.59)	2 (0.44)	2 (0.21)	Ref	Ref	-	-
Normal	267 (26.05)	66 (14.51)	100 (10.54)	0.74 [0.15,3.76]	1.12 [0.22,5.66]		
Overweight	426 (41.56)	196 (43.08)	371 (39.09)	1.38 [0.28,6.9]	2.61 [0.52,13.02]		
Obese	326 (31.8)	191 (41.98)	476 (50.16)	1.76 [0.35,8.8]	4.38 [0.88,21.84]		
**Smoking Status**							
Yes	51 (4.97)	34 (7.47)	60 (6.3)	1.54 [0.99,2.42]	1.28 [0.87,1.89]	1.74 [1.09,2.79] *	1.44 [0.94,2.22]
No	975 (95.03)	421 (92.53)	893 (93.7)	Ref	Ref	Ref	Ref
**Alcohol consumption**							
Yes	24 (2.34)	9 (1.98)	32 (3.36)	0.84 [0.39,1.83]	1.45 [0.85,2.48]	-	-
No	1002 (97.66)	446 (98.02)	921 (96.64)	Ref	Ref		
**Media Exposure**							
Yes	896 (87.33)	401 (88.13)	875 (91.82)	1.08 [0.77,1.51]	1.63 [1.21,2.19] *	1.18 [0.80,1.73]	1.52 [1.08,2.15] *
No	130 (12.67)	54 (11.87)	78 (8.18)	Ref	Ref	Ref	Ref
**Diet**							
High fat	217 (21.15)	97 (21.32)	218 (22.88)	1.01 [0.77,1.32]	1.11 [0.89,1.37]	1.13 [0.85,1.50]	1.25 [0.98,1.60]

Low fat	809 (78.85)	358 (78.68)	735 (77.12)	Ref	Ref	Ref	Ref
**Comorbidities**							
None	897 (87.43)	376 (82.64)	744 (78.07)	Ref	Ref	Ref	Ref
1 or more	129 (12.57)	79 (17.36)	209 (21.93)	1.46 [1.08,1.98] *	1.95 [1.54,2.48] **	1.39 [1.01,1.91] *	1.69 [1.30,2.20] **

Abbreviations: RRR, Relative Risk Ratio; CI, Confidence Interval, Ref, Reference Category; BMI, Body Mass Index
Crude RRR^1 = Partial Treatment versus No Treatment; Crude RRR^2 = Full Treatment versus No Treatment;
$aRRR^1$ = Partial Treatment versus No Treatment; $aRRR^2$ = Full Treatment versus No Treatment;
* P < 0.05, ** P < 0.001
Model Goodness of Fit, P = 0.928, AIC = 4632.86, BIC = 4806.78

Table 3: **Factors associated with DM-HTN-abdominal obesity among high-risk young and middle-aged individuals***

Variables	Previously diagnosed DM-HTN-High waist (N = 2434)		Newly diagnosed DM-HTN-High waist (N = 679)		Total DM-HTN-High waist (N = 3113)	
	**Crude RR (95% CI)**	**aRR (95% CI)**	**Crude RR (95% CI)**	**aRR (95% CI)**	**Crude RR (95% CI)**	**aRR (95% CI)**
**Age (years)**						
15-30	Ref	Ref	Ref	Ref	Ref	Ref
31-40	2.38 [2.07,2.74] **	2.11 [1.52,2.94] **	5.09 [3.69,7.01] **	4.26 [3.08,5.91] **	2.75 [2.42,3.12] **	2.52 [1.89,3.37] **
41-49	5.43 [4.77,6.19] **	6.03 [4.43,8.20] **	8.78 [6.43,11.99] **	7.24 [5.23,10.02] **	5.84 [5.18,6.57] **	6.12 [4.64,8.07] **
**Sex**						
Male	Ref	Ref	Ref	-	Ref	Ref
Female	3.21 [2.61,3.95] **	2.88 [2.16,3.84] **	1.24 [0.96,1.61]		2.40 [2.04,2.82] **	2.22 [1.76,2.82] **
**Education**						
No education	Ref	Ref	Ref	Ref	Ref	Ref
Primary	1.22 [1.07,1.4] *	0.83 [0.57,1.19]	1.28 [1.02,1.61] *	1.25 [0.99,1.57]	1.24 [1.10,1.39] **	0.88 [0.65,1.19]
Secondary	1.10 [1,1.22]	1.14 [0.87,1.49]	0.86 [0.71,1.03]	0.95 [0.77,1.16]	1.04 [0.95,1.14]	1.10 [0.88,1.39]
Higher	1.11 [0.97,1.27]	1.31 [0.94,1.82]	0.46 [0.34,0.62] **	0.56 [0.40,0.79] *	0.94 [0.84,1.06]	1.11 [0.83,1.49]

**Occupation**						
With higher physical activity	Ref	Ref	Ref	-	Ref	Ref
With lower physical activity	1.78 [1.47,2.15] **	1.09 [0.88,1.34]	1.08 [0.80,1.46]		1.55 [1.32,1.82] **	1.05 [0.88,1.26]
**Place of Residence**						
Rural	Ref	Ref	Ref	Ref	Ref	Ref
Urban	1.71 [1.58,1.86] **	1.15 [0.94,1.42]	1.73 [1.49,2.02] **	1.56 [1.31,1.86] **	1.71 [1.59,1.83] **	1.23 [1.03,1.47] *
**Wealth Index**						
Poorest	Ref	Ref	Ref	Ref	Ref	Ref
Poorer	1.51 [1.22,1.86] **	1.40 [0.80,2.46]	1.42 [1.02,1.97] *	1.18 [0.85,1.63]	1.48 [1.24,1.77] **	1.55 [0.98,2.47]
Middle	2.24 [1.84,2.73] **	1.94 [1.14,3.31] *	1.82 [1.34,2.48] **	1.23 [0.90,1.68]	2.11 [1.79,2.49] **	2.00 [1.29,3.11] *
Richer	3.14 [2.6,3.79] **	2.02 [1.19,3.45] *	1.89 [1.39,2.56] **	1.09 [0.79,1.51]	2.75 [2.34,3.22] **	1.99 [1.28,3.10] *
Richest	3.96 [3.29,4.77] **	2.59 [1.51,4.47] *	1.93 [1.42,2.61] **	0.96 [0.68,1.37]	3.33 [2.84,3.89] **	2.41 [1.53,3.80] **
**BMI (kg/m^2)**						
Underweight/ Normal	Ref	Ref	Ref	-	Ref	Ref

Overweight	1.74 [1.55,1.94] **	1.52 [1.16,1.99] *	2.40 [1.90,3.03] **	2.33 [1.83,2.95] **	1.85 [1.67,2.04] **	1.85 [1.45,2.35] **
Obese	5.69 [5.08,6.37] **	4.79 [3.65,6.28] **	7.86 [6.23,9.92] **	7.00 [5.47,8.94] **	5.97 [5.40,6.60] **	5.76 [4.51,7.36] **
**Smoking Status**						
Yes	0.53 [0.45,0.63] **	0.87 [0.62,1.22]	0.78 [0.60,1.02]	-	0.58 [0.51,0.67] **	0.79 [0.60,1.05]
No	Ref	Ref	Ref		Ref	Ref
**Alcohol consumption**						
Yes	0.48 [0.37,0.61] **	1.22 [0.84,1.78]	0.89 [0.63,1.26]	-	0.57 [0.46,0.69] **	1.40 [1.05,1.87] *
No	Ref	Ref	Ref		Ref	Ref
**Media Exposure**						
Yes	1.66 [1.46,1.89] **	1.87 [1.10,3.19] *	1.09 [0.89,1.35]		1.50 [1.34,1.67] **	1.53 [1.01,2.33] *
No	Ref	Ref	Ref	-	Ref	Ref
**Diet**						
High fat	1.31 [1.19,1.44] **	0.98 [0.78,1.24]	1.02 [0.84,1.24]		1.25 [1.14,1.36] **	1.04 [0.85,1.26]
Low fat	Ref	Ref	Ref	-	Ref	Ref
**Additional Comorbidities**						

None	Ref	Ref	Ref	Ref	Ref	Ref
1 or more	2.78 [2.50,3.08] **	1.58 [1.22,2.05] *	1.33 [1.02,1.73] *	0.90 [0.69,1.18]	2.44 [2.21,2.68] **	1.49 [1.18,1.88] *
**Model Fitness**						
AIC	-	4872.263	-	8756.782	-	6309.822
BIC	-	5054.068	-	8914.211	-	6491.628

Abbreviations: RR, Rate Ratio; aRR, Adjusted Rate Ratio; CI, Confidence Interval; Ref, Reference Category; DM, Diabetes Mellitus; HTN, Hypertension; BMI, Body Mass Index

* P < 0.05, ** P < 0.001

[a]Denominator (n = 267517) taken as individuals with higher-risk of metabolic syndrome, i.e., detected with DM or HTN or high waist or high BMI (≥25.0)

Figure 1

Click here to access/download;Figure;Screenshot 2023-08-24 204725.tiff

Figure 1: Health outcomes of participants with DM-HTN comorbidity along with High waist circumference

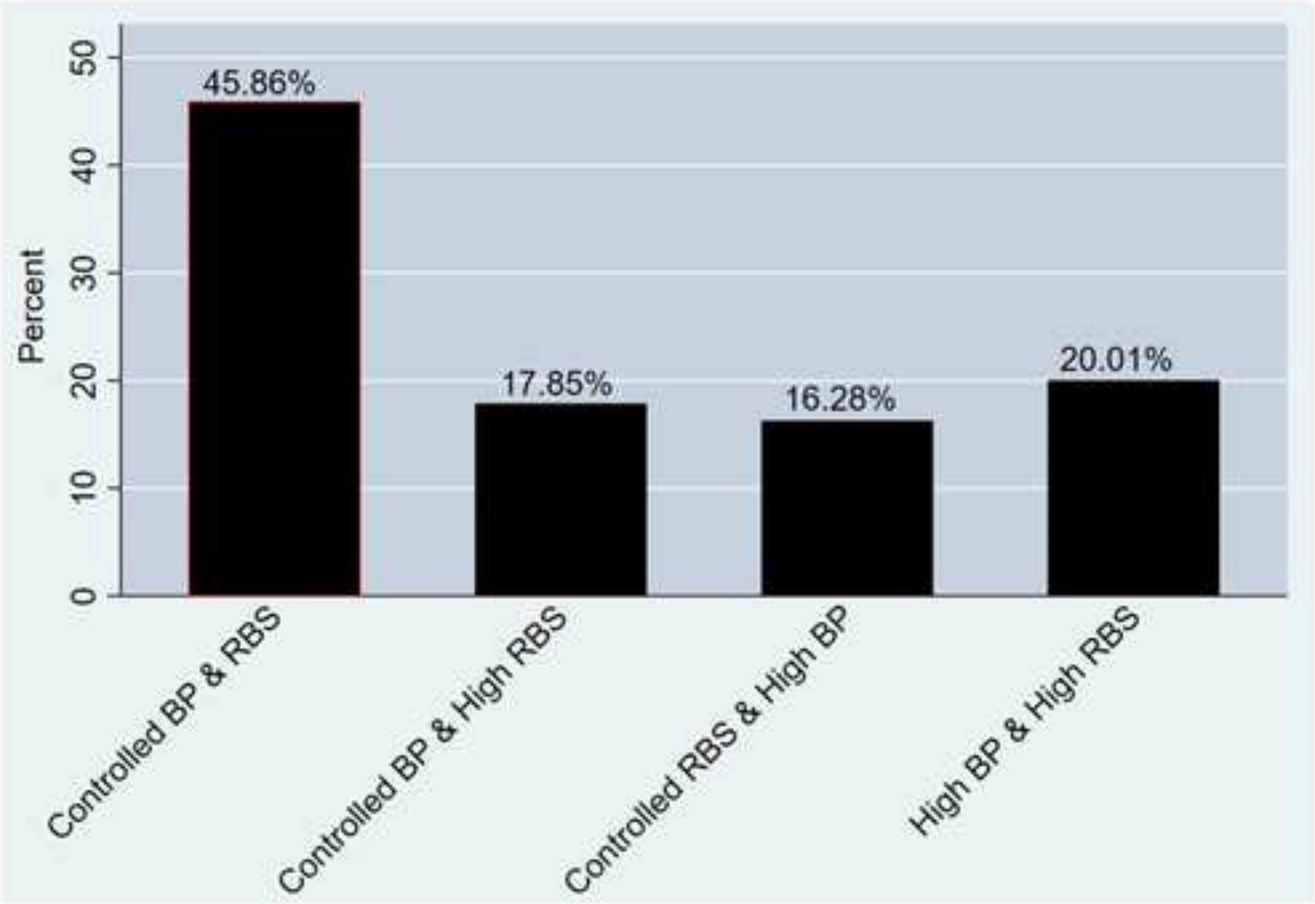

Figure 2

Click here to access/download;Figure;Screenshot 2023-08-24 204742.tiff

Figure 2: Prevalence of DM-HTN-Abdominal obesity in high-risk young and middle-aged persons in India

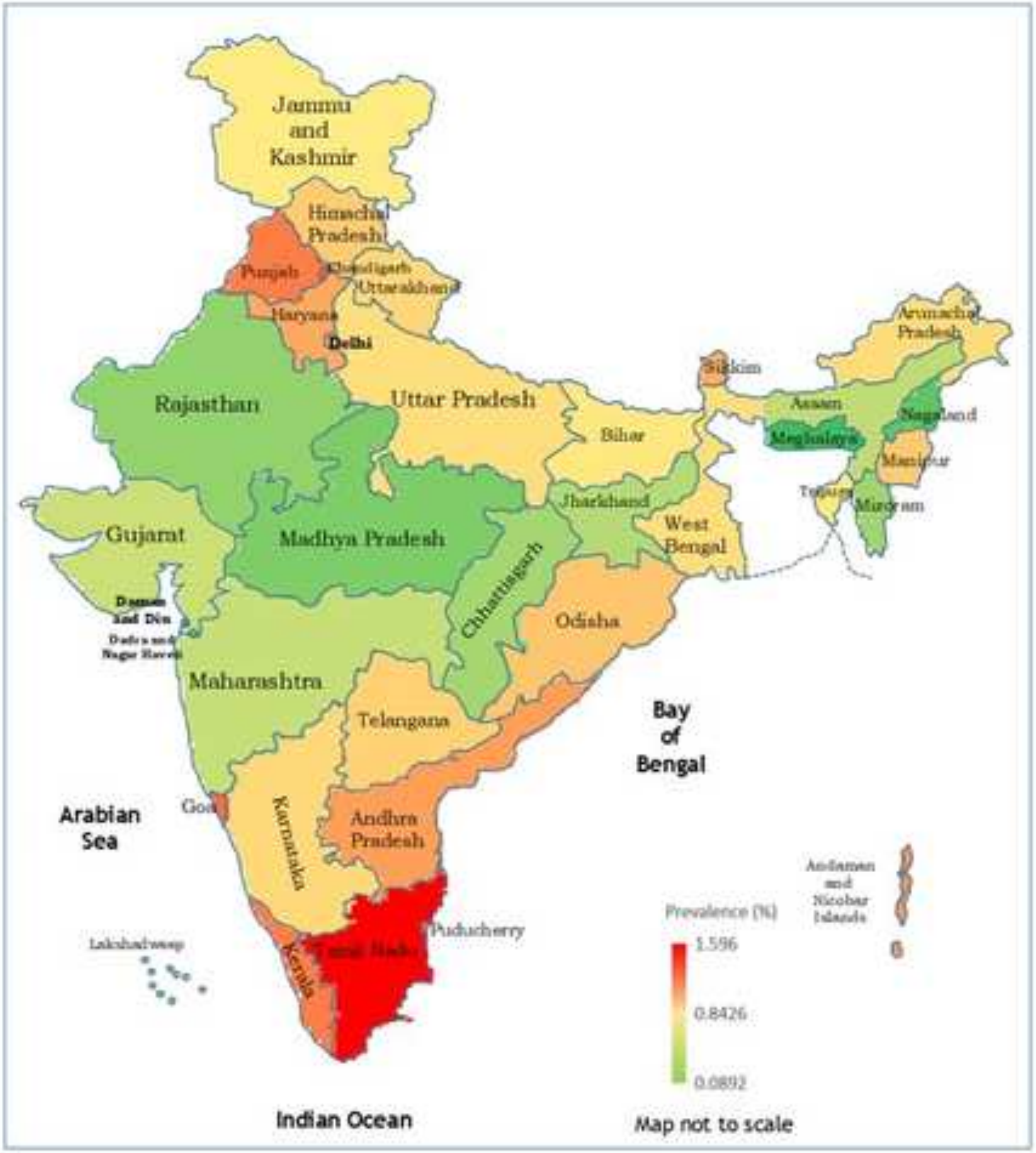

Declaration of Interests

Click here to access/download
Supporting Information
declaration-of-competing-interests.docx
