# Supplementary material for: The burden and care cascade in young and middle-aged patients with diabetes hypertension comorbidity with abdominal obesity in India: A nationally representative cross-sectional survey
Source: PLOS Glob Public Health. 2024 Jul 17;4(7):e0003413. doi: 10.1371/journal.pgph.0003413 (PMC11253957; doi:10.1371/journal.pgph.0003413)
Supplement: Attachment — Submitted filename: Response to Reviewers.docx [file pgph.0003413.s004.docx]

**Response to Reviewers**

***We thank the editor and reviewer for proving their valuable feedback and the opportunity to revise our manuscript***

| **Reviewer’s comments** | **Authors’ response** |
| --- | --- |
| 1. Valuable topic. A number of points need to be highlighted. | Thank you for the feedback. |
| 2. better to highlight only diabetes, hypertension, metabolic syndrome and India as keywords. | Complied. |
| 3. can you re-arrange the introduction as follow:  a. brief introduction or definition of the health problem in your topic.  b. directly give the prevalence and incidence globally and in the research area (if there is available data).  c. discuss the definition of DM, HT, metabolic syndrome and abdominal obesity as series of diseases in un separated idea. | Complied. |
| 4. is this the exactly age (18-49)? | Thank you for pointing this. We have corrected the typographical error (15-49 years). |
| 5. mention the instruction of data collection | Complied. We have added the information on the measured variable. |
| 6. define the standard level of the measured variable. | Cut-off for measurement of HTN and DM as per blood pressure and blood sugar levels are reported in the operational definitions. |
| 7. questionnaire date are represented as inclusion, exclusion criteria or instruction information to collect or to choose sample. | Complied. Inclusion and exclusion criteria have now been mentioned. |
| 8. Re-strained your methods to include Study design and area, Study subjects, Inclusion criteria, Exclusion criteria, methods for data Collection and Preparation of Samples. | Complied. The subheadings have been provided. |
| 9. set your participants in methodology as groups i.e; diabetic hypertensive, diabetic hypertension with abdomen obesity or with metabolic syndrome. | Complied. The outcome variable details have been expanded for improved clarity. |
| 10. criteria of disease+criteria of participant. you must set it for one time in inclusion and inclusion criteria of participants then you can write the standard criteria of your collecting your sample. | All the participants irrespective of their medical history underwent blood pressure and blood sugar estimations. This has been emphasized in the methods sections. |
| 11. write about the methods of analysis. you can describe the method in the text of table of results briefly. | Statistical analysis section has been elaborated. Method of measurement of blood pressure and blood glucose has been explained in detail in the methodology section. |
| 12. mean SD is not recorded. please add or insert table that include the average mean of variables. | Complied. We have now reported the mean and SD estimates of continuous variables under Supplementary Table 2. |
| 13. can the author determine the time of affecting by DM and HT, or what was the firstly diagnosed, DM Or HT. | Since no data is available in the original NFHS-5 datasets pertaining to the time of diagnosis of DM or HTN, or first diagnosis, we are unable to report these estimates here. We have now acknowledged this as a limitation. |
| 14. please point to the table which indicate the criteria of metabolic syndrome. | Complied. We have added the criteria as Supplementary Table 1. |
| 15. Divide your discussion into sub-title include: - conclusion - recommendations - limitation of the study - Acknowledgements. | Complied accordingly. |
