# Supplementary material for: The burden and care cascade in young and middle-aged patients with diabetes hypertension comorbidity with abdominal obesity in India: A nationally representative cross-sectional survey
Source: PLOS Glob Public Health. 2024 Jul 17;4(7):e0003413. doi: 10.1371/journal.pgph.0003413 (PMC11253957; doi:10.1371/journal.pgph.0003413)
Supplement: Attachment — Submitted filename: Response to Reviewers.docx [file pgph.0003413.s005.docx]

**Response to Reviewers**

***We thank the editor and reviewer for proving their valuable feedback and the opportunity to revise our manuscript***

| **Reviewer’s comments** | **Authors’ response** |
| --- | --- |
| **Reviewer 1:** |  |
| 1. thank you much for your response | Thanks for the feedback. |
| study design and data source: 2. Add the study design (cross sectional study) | Complied. |
| 3. reference is enough. author can delete the highlighted sentence. | Complied. |
| 4. better to start the paragraph by those points (re-arrange points). | Complied. |
| 5. better to classify the study participants in a table instead of text, according to specific criteria, for Diabetes mellitus, Hypertension and dyslipidemia. then add the source of classification below the table. | Complied and added accordingly as Supplementary Table 1. |
| 6. add the table and their source instead. | Complied. |
| 7. all questionnaire date which obtained from the respondent were included in the analysis. no need to duplicate information in different way. | Complied. We have now clarified the sentence accordingly. |
| **Reviewer 2:** |  |
| Congratulations on your efforts to analyse a very big data set with a specific research question that was adequately answered from your analysis. Please address the following comments that will strengthen the manuscript and enhance clarity: | Thank you for the feedback. |
| 1. Methodology: i) Was there a pre-study training exercise for the data collection research team? This need to be mentioned to give assurance that there was standardization of the data collected and guarantee reproducibility.  ii) Age was such an important outcome of the survey. Was it self-reported of objectively determined using official government documents like a passport, Birth Certificate, Driving licence, and related documents?  iii) Outside obtaining written consent to participate in the study please clarify if further consent to take waist circumference: this is only mentioned for BP and sugar measurements. This is important to ensure that the highest ethical standards were employed in the conduct of the study.  iv) The initial definition of DM is limiting (2nd sentence on page 10). though subsequent section in the manuscript clarify this further (correct definition). Please correct this to ensure consistency.  v) The observation that there were regional variations in the observed primary outcome of the study is hanging. What does this exactly mean?  vi) There is silence on how women who could have been pregnant were handled during the data collection. The female population studied were all in the bracket of childbearing potential. Was pregnancy an exclusion criterion? If so was it self reported on based on some documentation? | i) We have now added the relevant statement in support of the training of field staff.  ii) Age was “self-reported” by the participants and it has been clarified in the methodology  iii) Clarification added.  iv) Corrected. We thank the reviewer for pointing this out.  v) We meant to imply that there was significant geographic variation in the prevalence of our primary outcome across states and union territories across India as evident from our spot map. We have now commented on this point in the discussion.  vi) Thanks for pointing this out. Pregnant women (based on self-report) were excluded from the analysis. We have now added the relevant statement to the section on study participants. |
| 2. Discussion section: i) The number of undiagnosed participants may sound statistically a small percentage; but clinically significant. This need to come out because it is important as already captured in your recommendation.  ii) The discussion point that more than half of those with the primary outcome were not on treatment need to be backed with evidence from literature that justifies if treatment interventions of this specific fairly young population yields positive cardiovascular outcomes (CV mortality, strokes, Heart Failure, Coronary events). | i) Added the relevant statements in the discussion section.  ii) Complied and added the required text. |
| Conclusion i) This is not strictly based on the data presented. First, the conclusion statement should be based on your study objectives/ questions namely: ascertain burden, determinants and care cascade as related to the primary outcome of the study. The conclusion statement starts with the last aspect; but again it has a factual error. Your data say that 19.4% of the total participants with the primary outcome of interest with prior diagnosis (of DM, Hypertension) were already on full treatment; while 34.715 were on partial treatment. The total is 53.85%; much more than the stated figure in the conclusion. Additionally, those with undiagnosed disease could not have been on treatment for obvious reason.  ii) The recommendation needs to be backed with more discussion as captured above (2 ii). | i) We agree, 19.4% of those with the outcome were on partial treatment and 34.71% on neither treatment. Hence, the total of 53.85% has now been rephrased as more than half.  ii) Complied. We have now reframed the recommendations. |
