# Supplementary material for: The burden and care cascade in young and middle-aged patients with diabetes hypertension comorbidity with abdominal obesity in India: A nationally representative cross-sectional survey
Source: PLOS Glob Public Health. 2024 Jul 17;4(7):e0003413. doi: 10.1371/journal.pgph.0003413 (PMC11253957; doi:10.1371/journal.pgph.0003413)
Supplement: Attachment — Submitted filename: Response to Reviewers.docx [file pgph.0003413.s007.docx]

**Response to Reviewers**

***We thank the editor and reviewers for proving their valuable feedback and the opportunity to revise our manuscript.***

| **Reviewer’s comments** | **Authors’ response** |
| --- | --- |
| **Reviewer 1:** |  |
| 1. thank you for your patience and response. | Thanks for the feedback on our manuscript. |
| Methodology: a. Methodology still need precise effort to be in the frame. | Complied. We have rearranged as per your feedback. |
| b. Rearrange and summarise the information to avoid repetition and maintain the integrity of the section. | Complied. We have rearranged as per your feedback. |
| c. See all the comments in the enclosed document. The final description of data which obtained from questionnaire must be described in points of one paragraph include all: 1. Personal information 2. Biochemical measurements and their cut-off points 3. Anthrobometric measurements their cut-off points. 4. Sociodemographic and clinical data 5. Life style. | Complied. We have resolved all comments of the enclosed document and improved the methodology section accordingly. |
| d. Avoid copying the questions and answers of the questionnaire. Give only the abstract of your experience. | We have provided the exact questions from the survey to ensure transparency and reproducibility of our study, and allowing readers to understand the specifics of the data collection process. |
| **Reviewer 2:** |  |
| Reviewer #2: My comments have been adequately addressed. Congratulations on your efforts! | Thank you for the feedback. |
